# Health sector employment: a tracer indicator for universal health coverage in national Social Protection Floors

**DOI:** 10.1186/s12960-015-0056-9

**Published:** 2015-08-31

**Authors:** Xenia Scheil-Adlung, Thorsten Behrendt, Lorraine Wong

**Affiliations:** International Labour Organization (ILO), Route des Morillons 4, CH-1211 Geneva 22, Switzerland

**Keywords:** UHC, Social protection, Health worker employment

## Abstract

**Background:**

Health sector employment is a prerequisite for availability, accessibility, acceptability and quality (AAAQ) of health services. Thus, in this article health worker shortages are used as a tracer indicator estimating the proportion of the population lacking access to such services: The SAD (ILO Staff Access Deficit Indicator) estimates gaps towards UHC in the context of Social Protection Floors (SPFs). Further, it highlights the impact of investments in health sector employment equity and sustainable development.

**Methods:**

The SAD is used to estimate the share of the population lacking access to health services due to gaps in the number of skilled health workers. It is based on the difference of the density of the skilled health workforce per population in a given country and a threshold indicating UHC staffing requirements. It identifies deficits, differences and developments in access at global, regional and national levels and between rural and urban areas.

**Results:**

In 2014, the global UHC deficit in numbers of health workers is estimated at 10.3 million, with most important gaps in Asia (7.1 million) and Africa (2.8 million). Globally, 97 countries are understaffed with significantly higher gaps in rural than in urban areas. Most affected are low-income countries, where 84 per cent of the population remains excluded from access due to the lack of skilled health workers. A positive correlation of health worker employment and population health outcomes could be identified. Legislation is found to be a prerequisite for closing access as gaps.

**Conclusions:**

Health worker shortages hamper the achievement of UHC and aggravate weaknesses of health systems. They have major impacts on socio-economic development, particularly in the world’s poorest countries where they act as drivers of health inequities. Closing the gaps by establishing inclusive multi-sectoral policy approaches based on the right to health would significantly increase equity, reduce poverty due to ill health and ultimately contribute to sustainable development and social justice.

## Background

For many years, public debates focusing on increasing health sector employment were often concerned with health expenditure and fiscal consolidation measures rather than highlighting the crucial role of health workers in moving towards universal health protection and coverage (UHC).

Today, health worker shortages are dramatic, and closing resulting gaps in UHC appears to be an insurmountable obstacle in many countries. Such gaps have intensified the impact of the Ebola outbreak in West African countries and became visible as social and economic shocks: thousands of Ebola victims suffered from the nearly complete absence of local health workers and had to rely on hastily arranged global support. Besides the impacts on human health, trade and tourism came to a complete standstill and slashed the already low-GDP growth and income of the population in the region.

There is little doubt that the global health worker shortage defines the limits of effective social protection in health and can be considered as one of the most important barriers to progress on UHC. This is due to the fact that the health workforce constitutes the “primary determinant of and a necessary condition for effective coverage” [1]. With the ageing of the global population – including its health workforce – the current shortages are expected to magnify in the near future if no adequate policies are taken to address the issues.

However, the impact of aggregated health worker shortages on UHC has rarely been quantified at the global, regional or national levels. The data presented in this article aim at the following:

- closing the data gap;
- using the International Labour Organization (ILO) Staff Access Deficit Indicator (SAD) as a tracer indicator revealing the extent of the population without coverage and access to health care due to the deficit in sufficient numbers of health workers;
- providing information for decision-makers on highest returns of investments when striving towards UHC; and
- contributing to the discussion of impacts of investments in health sector employment on health protection coverage and access.

This article refers to recent research [2] in the area of health protection coverage and in the context of national Social Protection Floors (SPFs). In SPFs, health protection is a principal component of social protection providing coverage through national health services, national and social health insurances as well as other health-financing mechanisms that are based on prepayment such as taxes, contributions and premiums.

SPFs – outlined in ILO Recommendation 202 (R202) and adopted by 185 countries in 2010 – consist of government guarantees to ensure (1) universal access to at least essential health care that meet the criteria of availability, accessibility, acceptability and quality (AAAQ) and (2) basic income support through social protection mechanisms [3].

When establishing UHC in the context of SPFs, some principles should be applied. They include equity based on entitlements prescribed by law, fair financing and access without financial hardship as well as coherence of health, social, economic and developmental policies to ensure sustainable progress. Thus, achieving UHC in the context of SPFs requires the following:

1. the existence of inclusive legislation resulting in universal access to health care;
2. *the availability of a sufficient number of skilled health workers to make quality services equally accessible to all in need*;
3. adequate funds allowing for UHC of at least essential quality health care; and
4. the affordability of services and financial protection to ensure accessibility for all, particularly to avoid access barriers and financial hardship due to excessive out-of-pocket payments (OOPs).

**Figure 1:**
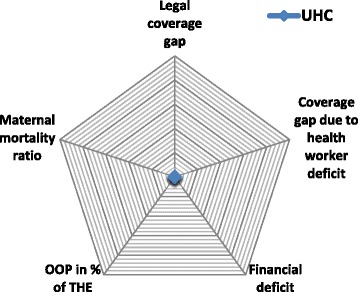
**Indicators for UHC in the context of SPFs [** 3 **].**

When assessing progress towards UHC in the framework of SPFs, all of these aspects need to be taken into account. Figure 1 provides an overview of the related indicators for UHC in the context of SPFs. AAAQ criteria are matched with indicators that are defined as deficits towards UHC: the legal health coverage deficit, the coverage gap due to health employment shortages and deficits in health spending (except OOP). Further, OOPs as per cent of total health expenditure (THE) are considered given the financial access barriers such payments create. In addition to these four indicators, the maternal mortality ratio (MMR) per 10 000 live births is used as a health systems’ outcome indicator.

Thus, SPFs focus on health sector employment as a key indicator for tracking progress towards UHC.

## Method

Health sector employment is a prerequisite for universal availability, accessibility and acceptability of quality services and maternal care requiring a sufficient number of skilled health workers – doctors, nurses and midwives – enjoying decent work. This includes adequate wage levels, skills development, occupational safety and health and others as outlined in the ILO Nursing Personnel Convention 149.

Against this background, the ILO SAD serves as a tracer indicator that informs about the share of the total population that has no access to health care due to the absence of skilled health workers. It refers to gaps in health sector employment towards UHC and identifies deficits, differences and developments in population coverage and access at the global, regional and national levels as well as between rural and urban areas.

The SAD is based on the difference between the density of the health workforce per population in a given country as indicated in the World Health Organization (WHO)’s Global Health Workforce Statistics [4] and a threshold representing the needed staffing requirements for UHC. The following formula is applied:

$$ \mathrm{SAD}=\left[\frac{\left(\mathrm{Threshold}-\mathrm{value}\kern0.5em \mathrm{country}\kern0.5em X\right)}{\mathrm{Threshold}}\times 100\right] $$

The threshold is crucial to help identifying the scope for improvement of understaffing, assessing the status quo and related performance towards UHC, optimizing investments in health sector employment and measuring progress.

The calculation of the threshold derives from the population-weighted median data of a group of countries determined by a set of criteria [5] including enabling health-financing mechanisms such as extent of out-of-pocket payments in total health expenditure and socio-economic conditions related to poverty and employment that facilitate adequate health sector employment needed for UHC. In 2015, the threshold amounts to 41.1 health workers per 10 000 population. It exceeds the minimum threshold identified by WHO in 2006 to provide the most basic health coverage rather than UHC by 18.3 health workers per 10 000 population [5]. Given the high correlation observed between skilled birth attendance (SBA) and health sector employment, SBA as indicated in the related WHO database [6] is used as a proxy to estimate rural/urban discrepancies in health sector employment.

The authors are aware that the limitations of the methodology are manifold. Firstly, they reflect the impacts of the very scarce data which in some cases also raise questions on reliability. This particularly concerns available disaggregated data which are often incomplete and not comparable at the global, regional and national levels. For example, across countries, standards for nursing vary significantly in terms of tasks and responsibilities, which in turn lead to differences in needed ratios of nurses to doctors. Thus, only aggregated data on health worker deficits rather than disaggregated data by skill mix are used. Consequently, no conclusions can be drawn on the shortages of specific professions, and results should not be interpreted with a view to resource allocation towards one or another profession. Secondly, as we use WHO’s Global Health Workforce Statistics [4], the methodology does not allow differentiating between public and private employment. Thirdly, there are also some methodological challenges that most likely result in underestimating deficits in coverage and access: this relates to the use of SBA data that due to the high-donor support provided for maternal care is most likely indicating a better performance of health worker availability than in other areas. Nevertheless, the data presented are currently the only and best data that are available to estimate the impacts of health worker shortages on UHC.

When interpreting the results, it should be taken into account that health sector employment cannot replace a full assessment of gaps in countries. Such an assessment requires contextualized interpretation taking into account further aspects such as the implementation of rights to health, for example, with regard to funding and OOP as well as the root causes of gaps beyond the health sector, such as poverty levels and the extent of the informal economy.

## Results and discussion

### Global and regional health sector employment required to achieve UHC in SPFs

**Figure 2:**
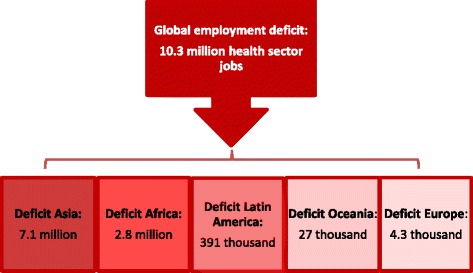
**Estimated number of health workers required to close global and regional gaps for UHC (ILO threshold 41.1 health workers per 10 000 population in 2014).**

Current health sector employment does not allow for access to health care for all in need. In 2014, estimated UHC deficits amount to 10.3 million health workers globally, with the most significant gaps in highly populated countries of Asia (7.1 million health workers) and many countries of Africa (2.8 million health workers) (Figure 2).

As a result, throughout all regions, 97 countries are understaffed and large shares of their population have no access to health care given the absence of skilled health workers [5].

The gaps are most prominent in rural areas. While currently about half of the world’s population is living in rural areas, only 23% of the global health workforce is employed in rural areas. In rural areas, health sector employment is short of 7.1 out of the 10.3 million missing workers [7].

Thus, achieving UHC and related health outcomes at the global level requires significant investments in the health workforce. Such investments have the potential to yield high economic returns in the form of gains in employment, productivity, economic growth and sustainable development, particularly in rural areas. Further, in times of economic and financial crises, investments in health protection contribute to socially responsible recovery and reduce poverty and inequalities [8].

In general, multiple financing options are available for investments in health sector employment aiming at achieving UHC. They range from reallocating current public expenditure, increasing tax revenues and health insurance contributions, borrowing or restructuring debts and/or using more accommodating macroeconomic frameworks that draw on developmental aid [5].

### Impact of staff deficits on health protection of the population

At the global, regional and national levels, the SAD – measuring the health protection coverage gaps towards UHC due to shortages of skilled staff – reveals massive access deficits and associated inequities in health protection impacting on large parts of the global population.

**Figure 3:**
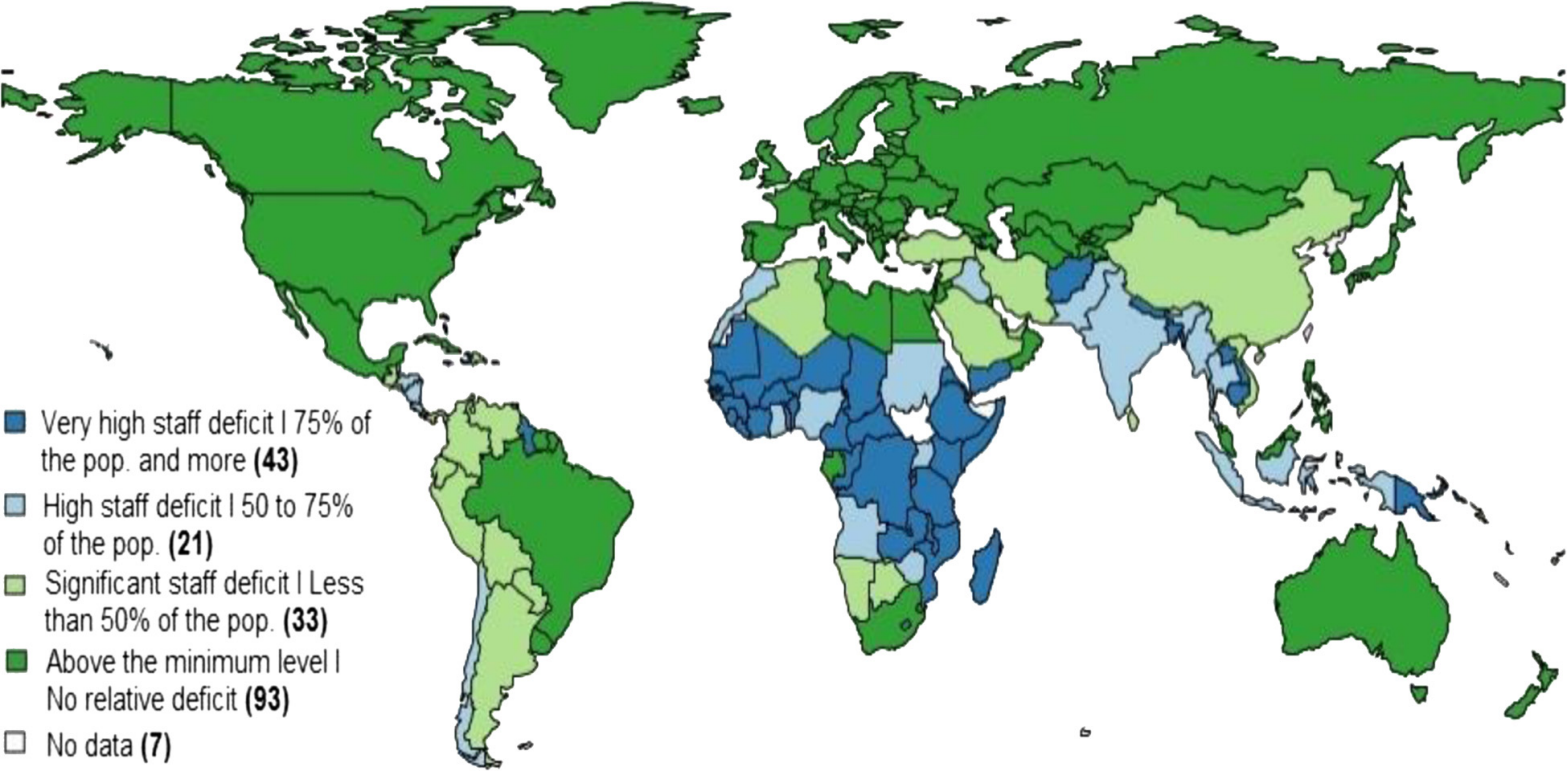
**Percentage of population globally not covered due to deficits in health workforce employment (per cent of population without access to quality health services in 2014).**
- Further, the SAD discloses that shortages in health coverage due to insufficient health worker employment are predominantly concentrated in low-income countries. Global deficits in health sector employment are thus concerning mostly the poorest countries of the world where many health systems are already weak. Due to these shortages, no health services are available for 84% of the population in low-income countries as compared to 23% in upper middle-income countries (Figure 4).

- When mapping the unavailability of quality services due to relative deficits in the health workforce, 64 countries are identified where more than 50% of the population has no health protection. In these countries, more than half of the population has no access to health care due to the lack of sufficient health sector employment (Figure 3). Investing in increased health sector employment in these countries would yield high benefits in minimizing global differentials in health protection and increase global equity in access to health care.

**Figure 4:**
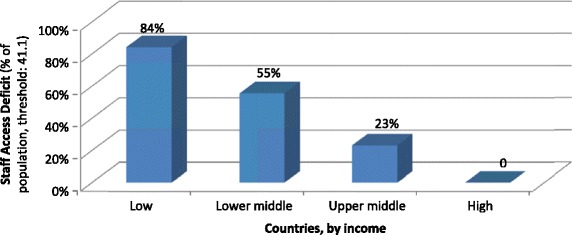
**Estimates of coverage gaps (in per cent of population) due to health workforce shortages, by income level of countries (ILO threshold 41.1 health workers per 10 000 population in 2014).**
- Thus, investments in health sector employment in low-income countries would ease the poorest parts of the global population from the burden of ill health and have the potential to indirectly reduce deepened or increased poverty.
- Globally, the highest shares of populations excluded from health care due to the unavailability of staff are found in the countries of Africa and Asia that have less than 3 health workers per 10 000 population: This is the case in Guinea where coverage and access deficits amount to 97.2% of the population. Similarly, high rates are found in Liberia and Sierra Leone – countries that have been most severely stricken by Ebola since the outbreak in 2014 (Table 1).

**Table 1:** **National deficits of skilled health workers and resulting population coverage gaps (data 2011 or latest available year)**

**Country**	**Number of health workers per 10 000 populations**	**SAD: estimated deficit in population coverage due to absence of health workers (in per cent of total population)**
Guinea	1.43	97.2
Niger	1.56	96.6
Sierra Leone	1.88	95.3
Liberia	2.88	94
Haiti	3.6	93.3
Mozambique	3.67	92.6
Senegal	4.79	89.4
Bangladesh	5.74	86.4

*Source*: ILO Social Protection Database 2014/2015 [5]

Other countries with SADs above 80% of the population include Niger, Haiti, Mozambique, Senegal and Bangladesh. In these countries, only between 1.56 and 5.74 health workers are available to deliver services to every 10 000 people. A lack of progress in health worker employment and UHC will further aggravate weaknesses of the health schemes and systems and will have major impacts on the development and socio-economic outcomes [9] besides the threats to health protection and access to most essential care.

### Impact of gaps in health sector employment on social and health outcomes

The SAD also reveals that gaps in health sector employment impact strongly on social outcomes, particularly inequities and indirectly poverty as well as health outcomes.

**Figure 5:**
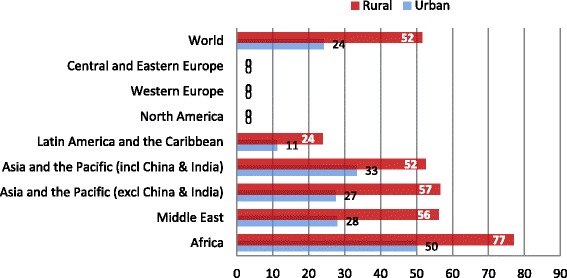
**Rural/urban coverage gaps due to staff access deficit, by region.**

Within countries, gaps in health sector employment as identified by the SAD constitute a major concern for health protection given the often inequitable distribution of health workers, particularly between rural and urban areas. In all regions of the world, the population living in rural areas is experiencing the highest access deficits to health care far from UHC. The uneven situation is illustrated by the fact that 52% of the global population living in rural areas as compared to 24% in urban areas is excluded from health services due to staff deficits (Figure 5). Concerned are particularly people living in Africa and Asia. Increased health sector employment in rural areas would not only reduce significantly inequities within countries but also result in reduced rural poverty given the close link between ill health and poverty.

**Figure 6:**
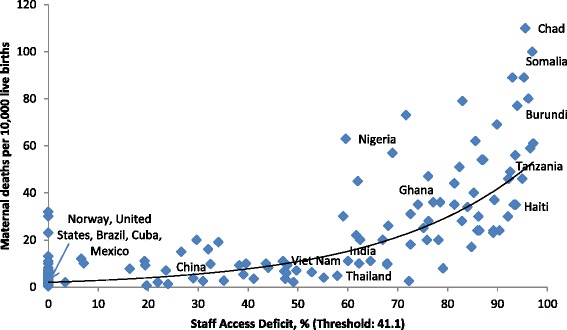
**Maternal mortality ratios and global gaps in health sector employment.**

An adequate health workforce is recognized to be crucial to improving population health outcomes [8]. Countries that have invested into their health workers such as Brazil, Ghana, Mexico and Thailand have also considerably improved the health status of their populations [9]. When assessing health sector employment and maternal mortality, a positive correlation of health workforce shortages and maternal mortality ratios is revealed (Figure 6).

The situation is worsened when differentiating between rural and urban areas and poorer and richer women as well as countries with lower and higher income levels [5]. Thus, closing gaps in health sector employment will improve life expectancy in countries and result in more equitable health outcomes among groups that are disadvantaged.

### Effects of rights-based health protection on health sector employment

**Figure 7:**
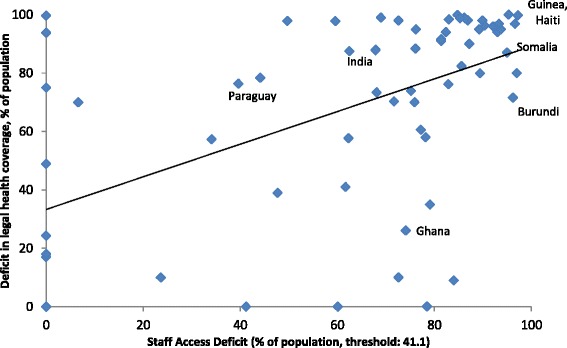
**Legal health protection and gaps in health sector employment in low- and lower middle-income countries.**

Figure 7 shows that in low- and lower middle-income country gaps in health sector employment are less significant if adequate levels of health protection are anchored in legislation (legal coverage). Thus, rights-based approaches for health protection, such as legislation or social health insurance contracts, contribute to closing gaps towards UHC in health sector employment. In fact, countries protecting their population by rights tend to employ more health workers than countries with fragmented, limited or no rights-based approaches. Hence, investments in health sector employment based on legislation for UHC are most likely to be more efficient and effective for broad parts of the population than investments that are undertaken without implementing the right to health for all.

## Conclusions

### Tracing deficits and challenges in UHC and access

Using health sector employment, particularly the SAD, as a tracer for UHC discloses the most important gaps and challenges in health protection coverage and access to health care:

– the quantitative deficit in numbers of health workers needed to achieve UHC at the global, regional and national levels;

– the share of the population lacking health protection and access to care due to gaps in health sector employment at the global, regional and national levels; and

– the extent of inequities in health care access of populations living in countries with different income and poverty levels as well as with regard to rural/urban disparities.

Based on the estimates, we conclude that the global, regional, national and subnational gaps in health sector employment weaken the availability, affordability, accessibility and quality of health care services and result in access barriers and impoverishment, particularly in rural areas. Further, social outcomes of health worker shortages point to the fact that related UHC gaps can be considered as drivers of health inequities. In addition, the SAD allows concluding that non-addressing gaps in health sector employment result in higher mortality and increased economic costs of ill health.

The results provided inform policies aiming at achieving UHC in the context of SPFs on qualitative and quantitative impacts of (not) realizing the right to health of the population. However, the estimates presented should be complemented by additional analyses on the needed skill mix of the health workforce as well as a full assessment based on the AAAQ criteria. This requires taking further aspects and indicators into account, mainly revealing gaps in legal coverage and financing deficits, as well as deficits in the affordability of services and financial protection due to OOP.

### Directing investments towards areas of high impact

The estimates presented identify areas where investments in higher health sector employment – if embedded in UHC/SPF policies – could achieve the greatest impacts and returns in terms of social, health and economic outcomes.

This is particularly the case in low-income countries of Africa and Asia and globally in rural areas. It would be the most rewarding to expand health protection coverage and develop inclusive approaches focusing on effective access to health care in these countries. Such investments have the potential to:

- realize human rights to health and social security
- increase equitable access to health care and thus equity in health
- reduce poverty and impoverishment
- contribute to economic growth
- sustain development by increased employment and productivity
- result in social peace, social justice and cohesion.

### Revealing the necessity for aligned multi-sectoral policies for progress towards UHC in SPFs

The results presented reveal the complexity and multiple dimensions involved in achieving UHC and in meeting the AAAQ criteria. They also reflect the need for specific policies both within and beyond the health sector.

Developing and implementing inclusive legislation on UHC within SPFs is a prerequisite that is beneficial for progress in developing the health workforce. Consequently, vertical health funding focusing on, for example, one specific disease, is less conducive than overall health system development.

In addition, focusing on enabling labour market policies is crucial. While governments do not have full control of labour markets for health workers, it is important to ensure that regulations aiming at equity in access to at least essential health care are in place. Thus, governments should ensure that health sector employment is not guided by fiscal constraints and improve the distribution of the health workforce in rural and urban areas. Further, improved international cooperation in the area of migration of health workers should be considered.

Likewise, investments in training, skill development and employment conditions of health workers, including adequate wages and incentives as well as enabling working conditions ranging from occupational safety and health to part-time work, are of key importance. This concerns particularly employment in the public sector if working conditions are less attractive than in the private sector. Besides policies that increase retention rates due to better working conditions, it may also be necessary to regulate the private sector with a view to ensure equity in access [10].

Further, best use of skills is important so as to ensure services of highest quality as well as the most efficient and effective performance of the scarce health workforce. This requires better matching health and social protection schemes and systems, related institutions and financing mechanisms as well as redefining boundaries and shifting responsibilities of health, social and domestic workers as well as family carers, for example, in cases of long-term care.

Finally, achieving sustainability and maximizing the impact of investments require the alignment and coordination of health, social, economic and developmental polices in order to alleviate poverty and to transform informal labour markets and other informalities that negatively impact. Thus, health policies need to be embedded in broader social (protection) policies. At the national level, this requires the development and implementation of inclusive legislation on Social Protection Floors providing financial protection and access to affordable quality health services that are available. At the global level, the post-2015 agenda needs to focus on closing deficits in the health workforce to achieve UHC in the context of SPFs.

## Abbreviations

AAAQ: Availability, Accessibility, Acceptability, and Quality (criteria)
ILO: International Labour Organization
MMR: Maternal Mortality Ratio
R202: ILO Recommendation 202 concerning national floors of social protection
SAD: Staff Access Deficit Indicator
SPFs: Social Protection Floors
UHC: Universal Health Coverage
WHO: World Health Organization
